# Supplementary material for: A plastid carbohydrate carrier mediates ribose recycling from nucleotide catabolism and glucose export from starch degradation
Source: Nat Commun. 2025 Nov 5;16:9747. doi: 10.1038/s41467-025-65510-8 (PMC12589546; doi:10.1038/s41467-025-65510-8)
Supplement: Supplementary file 1 — Supplementary Information [file 41467_2025_65510_MOESM1_ESM.pdf]

A plastid carbohydrate carrier is involved in ribose recycling from nucleotide catabolism and glucose export from starch degradation

Luisa Voß, Isabel Keller, Rebekka Schröder, Denise Mehner-Breitfeld, André Specht, Gerald Dräger, Jannis Rinne, Jakob Franke, Nieves Medina-Escobar, Marco Herde, Thomas Brüser, H. Ekkehard Neuhaus, Claus-Peter Witte

SUPPLEMENTARY INFORMATION

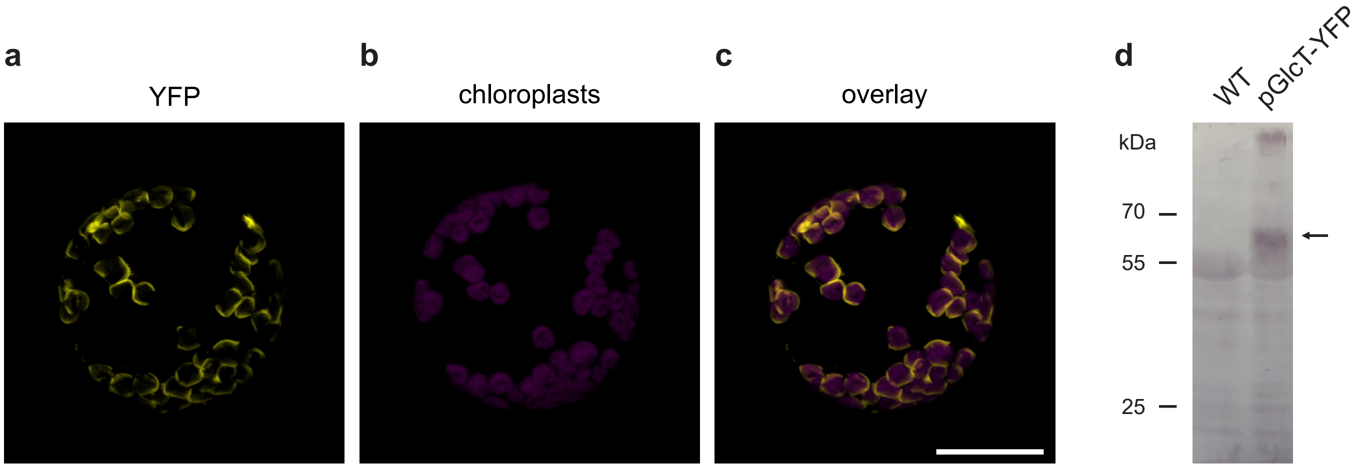

Supplementary Fig. 1 Mesophyll protoplast isolated after transient expression of *At*-pGlcT-YFP in leaves of *Nicotiana benthamiana*.

Confocal fluorescence microscope images of a mesophyll protoplast isolated from leaves of *N. benthamiana* transiently expressing *At-pGlcT-YFP* from the construct H391. **a** YFP channel, **b** chloroplast auto-fluorescence, **c** overlay of images in a and b. Scale bar, 7.5 µm. **d** Immunoblot of 10 µL clarified protein extract from *At-pGlcT-YFP*-expressing and wild type control *N. benthamiana* leaves. A monoclonal anti-GFP antibody from mouse (Roche 11814460001, clones 7.1 and 13.1, 1:5000 diluted) and a goat anti-mouse IgG antibody conjugated to alkaline phosphatase (Sigma A3562, lot no. SLCP2562, 1:30000 diluted) were used for detection of YFP.

The fusion protein *At*-pGlcT-YFP is detected with a molecular mass of about 68 kDa. The predicted size is 84 kDa indicating that the plastid transit sequence is cleaved after import. Consistently, Weber et al. (2000)[28] reported that the molecular mass of processed pGlcT is 43 kDa, which together with YFP (25 kDa) adds up to 68 kDa. The data show that the *At*-pGlcT-YFP fusion protein is likely fully processed and stable since no free YFP was detected on the immunoblot.

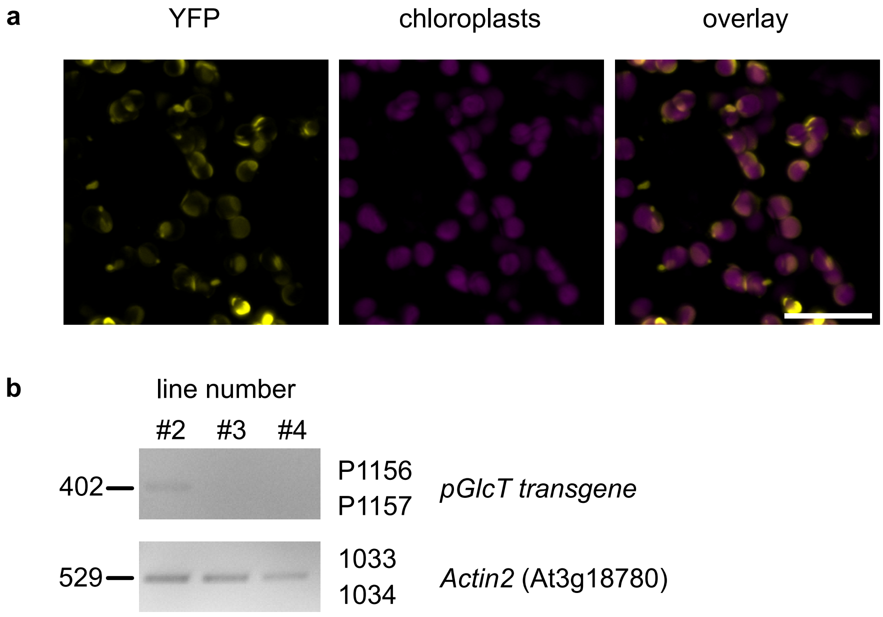

Supplementary Fig. 2 Analysis of *pglct* complementation lines expressing transgenes encoding *At*-pGlcT-YFP and untagged *At*-pGlcT.

a Confocal fluorescence microscopy images of leaf mesophyll from a transgenic Arabidopsis *pglct-#2* plant expressing *At-pGlcT-YFP* (construct H391, line #2). From left to right, YFP channel, chloroplast auto-fluorescence channel, and overlay of both channels. Scale bar, 20 µm. **b** RT-PCR result using RNA from transgenic Arabidopsis *pglct-#2* plants containing a *35S:At-pGlcT* transgene (construct H392, lines #2 to 4). A 402 bp product specific to the transgene was amplified with primers P1156 and P1157. A product was only obtained from RNA of line #2, which was used for further experiments. A 529 bp fragment was amplified from the transcript of *Actin2* (At3g18780) with primers 1033 and 1034 as control.

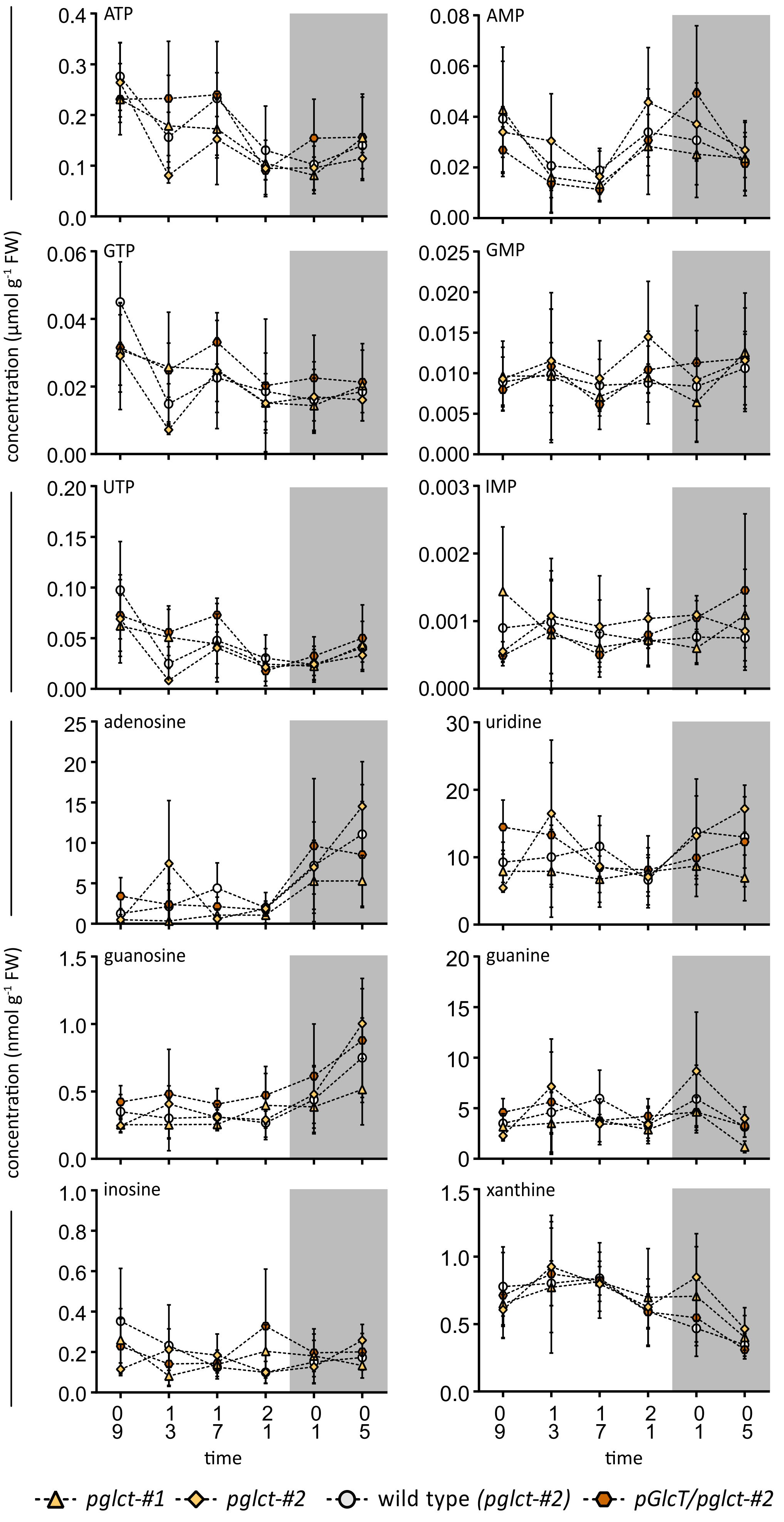

Supplementary Fig. 3 Diurnal changes in leaf nucleotide, nucleoside and nucleobase content of different *At-pGlcT* variants.

Data in this figure and in Fig. 2 are derived from the same plant material. Wild type (obtained from a segregating *pglct-#2* population), the two mutants (*pglct-#1, pglct-#2*) and the complementation line (*pGlcT/pglct-#2*) were analyzed in the four-leaf stage after 10 days of growth under long day conditions (16 h light). Mean values with SD are shown, n = 5. Each biological replicate (n) was a pool of seedlings grown on soil in an individual pot.

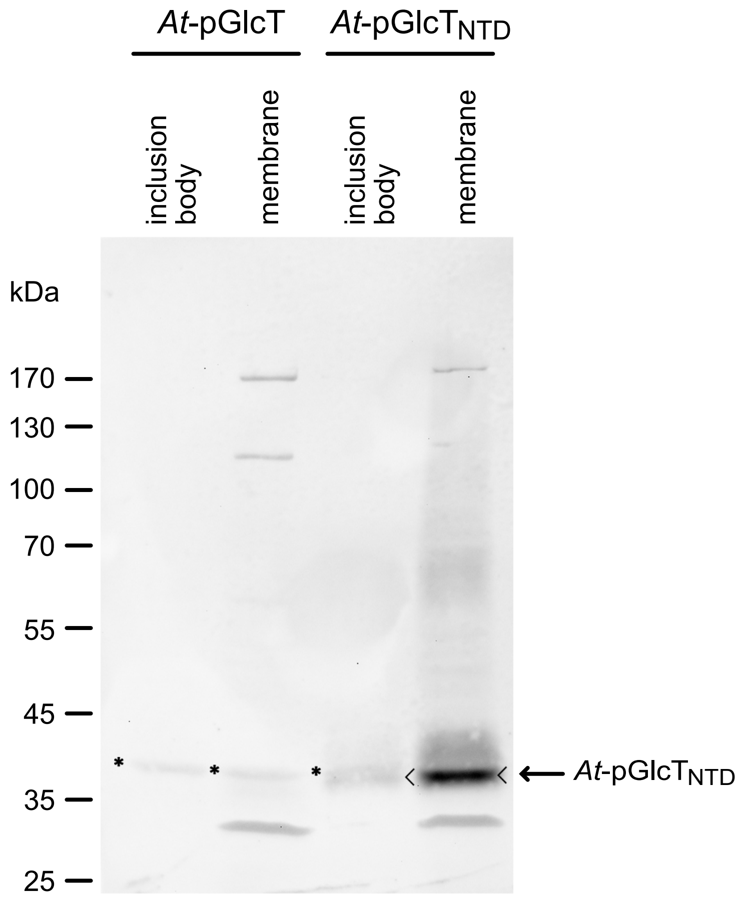

Supplementary Fig. 4 Immunoblot analysis of inclusion body and membrane fractions of *E. coli* cells expressing *At-pGlcT* or *At-pGlcT*$_{NTD}$.

E. coli cells produced either full-length *At*-pGlcT or *At*-pGlcT$_{NTD}$. Both proteins carried a C-terminal His-tag that was used for detection with an anti-His antibody. In *At*-pGlcT$_{NTD}$, the plastid transit peptide (residues 1 to 82) was replaced by the N-terminal region of the *E. coli* membrane protein PhoR[45] (residues 1 to 54) to foster membrane targeting.

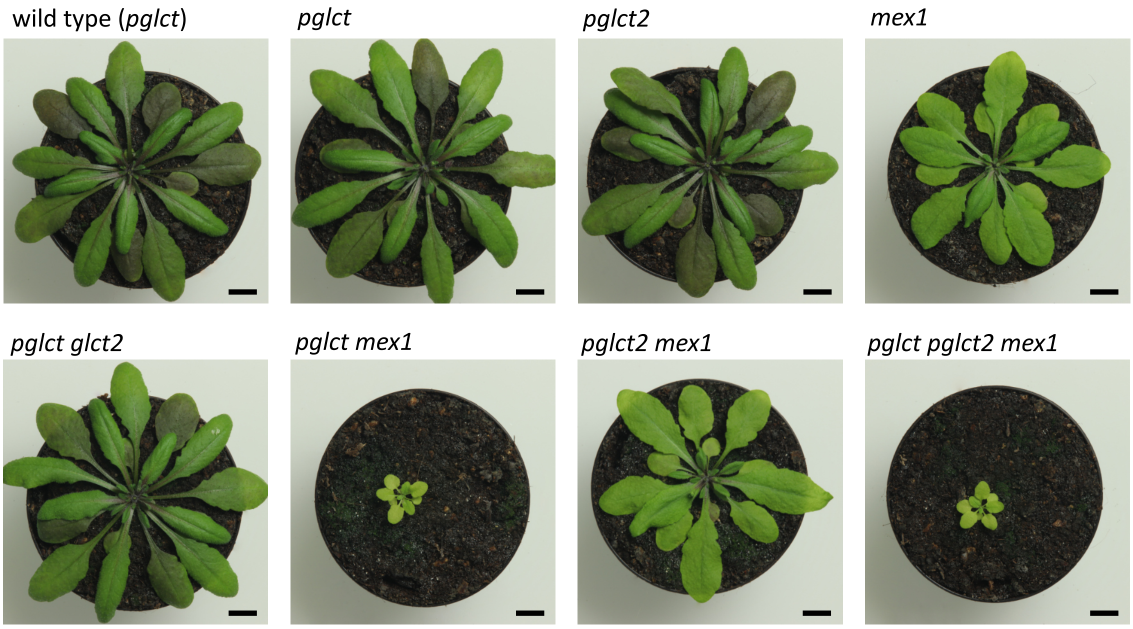

Supplementary Fig. 5 Images of 34-day-old Arabidopsis plants with single or combined defects in the two plastid glucose carriers (pGlcT, pGlcT2) and in the plastid maltose transporter (Mex1).
Representative images of 34-day-old plants of the indicated genotypes. This is a sample of the same plants that were analyzed after 12 days of growth (Fig. 5). Scale bar, 1 cm.

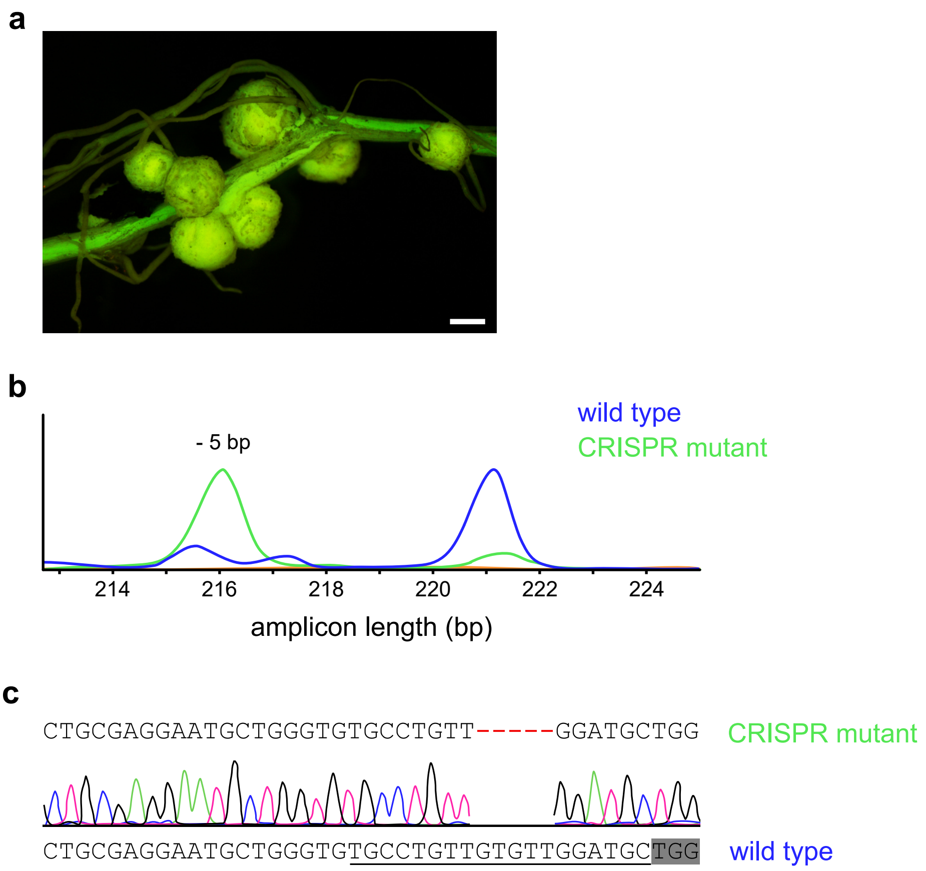

Supplementary Fig. 6 Analysis of *Phaseolus vulgaris* nodule mutants.

a *P. vulgaris* transgenic hairy root with nodules expressing a CRISPR mutagenesis construct that also encodes green fluorescent protein (GFP) as selection marker. The image was taken with a Nikon SMZ25 fluorescence binocular equipped with a GFP filter (Nikon P2 GFP-L). Scale bar, 0.5 cm. **b** Example chromatogram of an Amplified Fragment Length Polymorphism (AFLP) analysis using nodule DNA from wild type plants (WT, blue) and a homozygous CRISPR-induced mutant in *RBSK* (green). The DNA fragments were amplified by PCR with fluorescent labeled primers. The PCR amplicons were separated according to their size and detected using a capillary DNA sequencer (Applied Biosystems Prism 310). The amplicon length was estimated using a size marker. In this example, a deletion of 5 bp had occurred in both alleles resulting in only one signal for the mutant. **c** Sanger sequencing result using the DNA of the mutant shown in b confirming a homozygous 5 bp deletion (dashes). The predicted gRNA binding site is underlined and the protospacer adjacent motif (PAM) highlighted by a grey box in the wild type sequence.

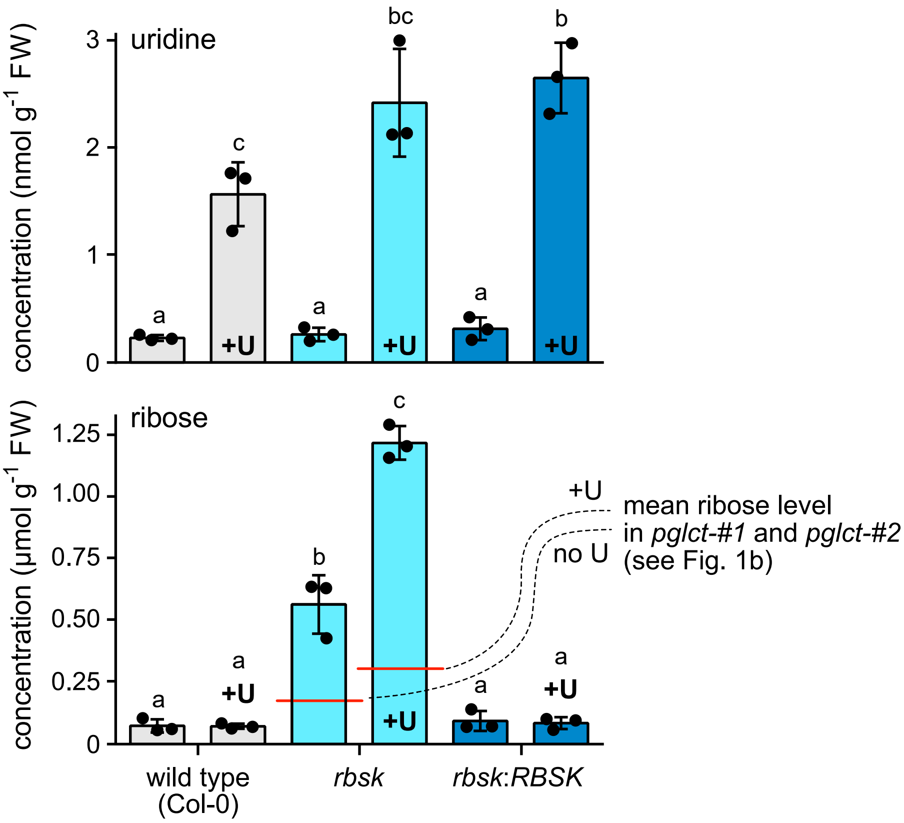

Supplementary Fig. 7 Ribose and uridine contents in seedlings of Arabidopsis *RBSK* variants before and after uridine addition.

Uridine and ribose contents in seedlings of an Arabidopsis *RBSK* mutant (*rbsk-1,* SALK007531) and a complementation line *rbsk-1:RBSK-HAStrep* (Schröder et al., 2018)[14] grown in a liquid shaking culture under continuous light for seven days followed by 24 hours of cultivation in new growing media containing 410 µM uridine (labeled +U in the graph) or control media without uridine. The mean ribose concentration in *pglct* seedlings (*pglct-#1* and *pglct-#2* together calculated with data from Fig. 1b) with and without uridine addition are indicted by red bars. Plants were thoroughly washed with distilled water before preparing the material for MS analysis. Error bars are SD, n = 3 biological replicates. Statistical analysis with two-sided Tukey's multiple pairwise comparison using the sandwich variance estimator. Different letters indicate significant differences at $p < 0.05$. The source data and all p values can be found in the Source Data file.

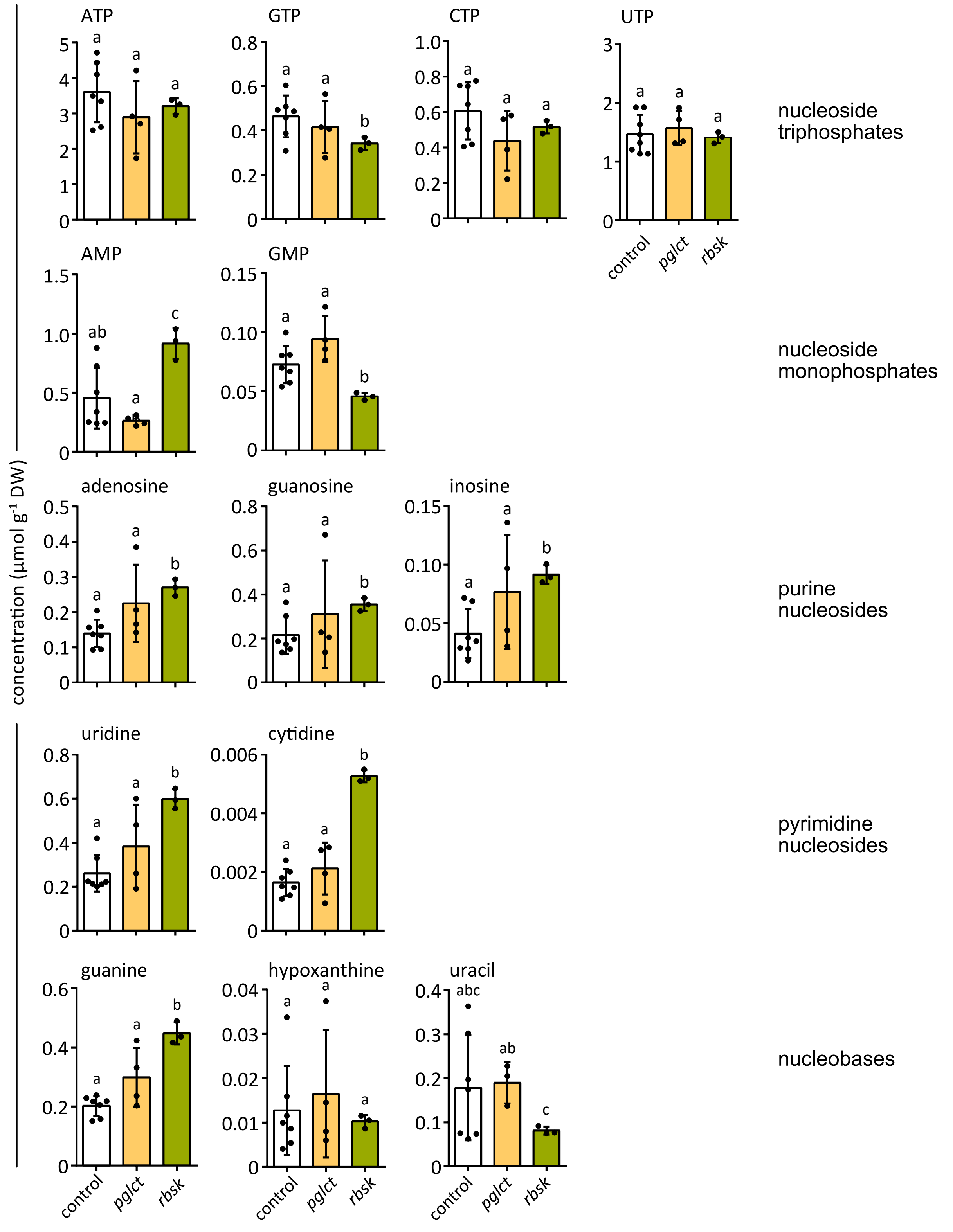

Supplementary Fig. 8 The content of nucleotide metabolism intermediates in bean nodules lacking pGlcT or RBSK.

This figure is an extension of Fig. 6 showing the content of further nucleotides, nucleosides and nucleobases not directly involved in ureide biosynthesis. The data in Fig. 6 and this data are derived from the same biological material.

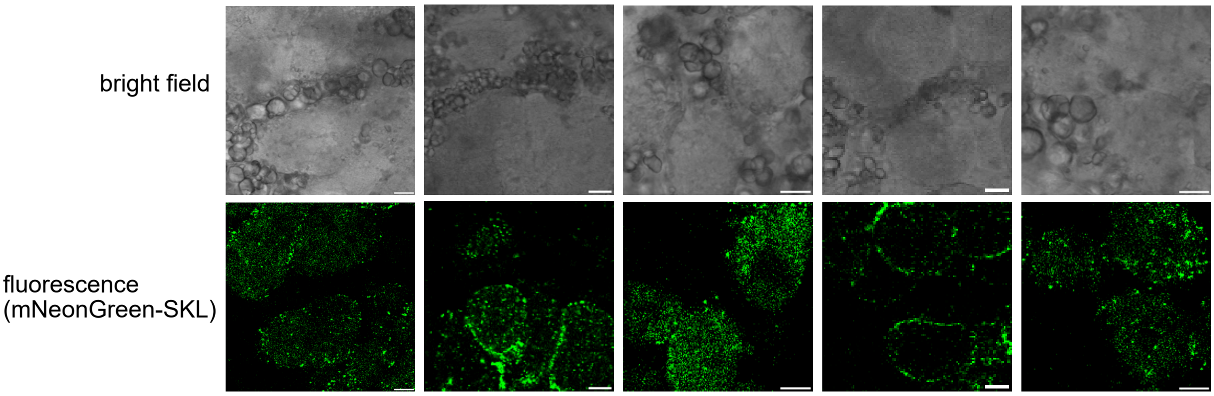

Supplementary Fig. 9 Activity of the *Pv-pGlcT;a* promoter of *P. vulgaris* in nodules visualized with close-up images of the infection zone

Close-up view of five independent nodule cross sections in the infection zone showing neighboring infected and uninfected areas with starch granules. The cross sections were obtained from two independent nodules. These images and the images in Fig. 7b were used for the quantification shown in Fig. 7c (see supplementary methods). Upper panels, bright field channel; lower panel, fluorescence channel. Scale bars, 10 µm.

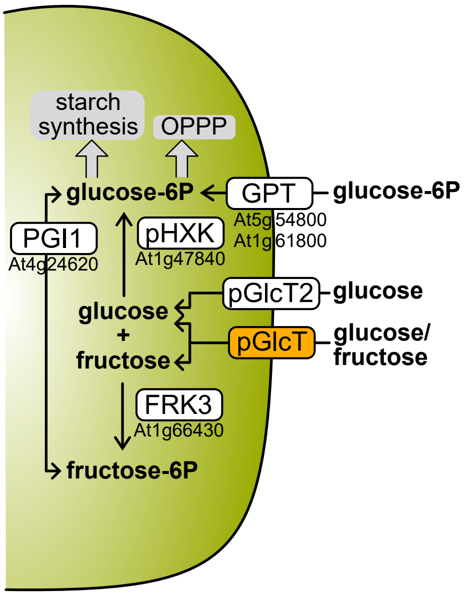

Supplementary Fig. 10 Model for a putative function of pGlcT in glucose and fructose import into plastids. Plastids possess a hexokinase (pHXK) and a fructokinase (FRK3) which can phosphorylate glucose and fructose, respectively. Phosphoglucoisomerase 1 (PGI1) interconverts fructose-6-phosphate and glucose-6-phosphate in plastids. Employing these plastid enzymes, imported glucose and fructose could be used in non-photosynthetic plastids to drive starch synthesis or the OPPP. However, direct import of glucose-6-phosphate by the glucose-6-phosphate phosphate antiporter (GPT) is known to be a main glucose-6P source in non-photosynthetic plastids.

Supplementary Table 1 Relative recovery of nucleotides, nucleosides and allantoin from nodule tissue. The sample matrix was generated from 1 mg of homogenized nodules. Recovery rates were calculated with isotope labeled metabolites. *n* = 3 technical replicates.

nucleotide	recovery (%)
ATP	74 ± 15
GTP	73 ± 18
CTP	75 ± 12
UTP	70 ± 23
GMP	86 ± 8
CMP	87 ± 2
IMP	77 ± 17
AMP	154 ± 33

nucleoside/base	recovery (%)
adenosine	58 ± 17
cytidine	111 ± 35
guanine	102 ± 38
guanosine	71 ± 7
inosine	74 ± 6
uracil	84 ± 5
uridine	86 ± 8
xanthine	87 ± 15

ureide	recovery (%)
Allantoin	65 ± 30

Supplementary Table 2 Overview of CRISPR-induced mutations in *Pv*-p*GlcT;a* and *Pv-RBSK* target genes of *P. vulgaris*. Samples were classified as chimeric if more than two type of mutations were detected for a gene and as incomplete if only one allele of a gene was edited.

	Control	*pGlcT;a*	*RBSK*
no. of samples	8	10	10
wild type	8	1	2
chimeric	0	0	1
incomplete	0	5	4
mutant	0	4	3

Supplementary Table 3 Detailed AFLP results of mutant nodules used for metabolite analysis. Genotypes of mutants whose metabolite profiles are shown in Fig. 6 and Supplementary Fig. 8. Only frame-shift mutations were found. The eight empty vector control nodule samples were also analyzed for mutations in *Pv-pGlcT;a* and *Pv-RBSK* but only wild type was detected.

mutant no.	*Pv-pGlcT;a*
mut 1	(-4 bp/ -4 bp)
mut 2	(+1 bp/ +1 bp)
mut 3	(+1 bp/ +1 bp)
mut 4	(+1 bp/ +1 bp)

mutant no.	*Pv-RBSK*
mut 1[1]	(-5 bp/ -5 bp)
mut 2	(-7 bp/ -4 bp)
mut 3	(-5 bp/ -1 bp)

[1] the detailed analysis data of this mutant are shown as example in Supplementary Fig. 6

Supplementary Table 4 Media compositions.

Composition of 5-fold concentrated M9 salts. The pH was adjusted to 7.5 with NaOH.

component	amount per liter	
Na_2HPO_4	30.0	g
KH_2PO_4	15.0	g
NH_4Cl	5.0	mL
NaCl	2.5	mL

M9 minimal media composition. Ribose or xylose was used as carbon source.

component	concentration		amount per liter	
M9 salts pH 7.5	5	X	200.0	mL
$MgSO_4 \times 7\ H_20$	1	M	2.0	mL
$CaCl_2 \times 2\ H_20$	1	M	0.1	mL
carbon source	20	% (w/v)	20.0	mL
Chloramphenicol (Cm)	25	mg mL^{-1}	1.0	mL
dH_20	-		776.9	mL

Supplementary Table 5 LC-MS parameters for allantoin and uric acid quantification. Retention times are affected by matrix and can vary.

MS source parameter	positive ion mode	negative ion mode
ion source	AJS ESI	AJS ESI
gas temperature	300°C	350°C
gas flow	12 L min^{-1}	12 L min^{-1}
nebulizer	30 psi	40 psi
sheath gas heater	300°C	350°C
sheath gas flow	11 L min^{-1}	12 L min^{-1}
capillary	4,000 V	2,500 V

analyte	ion mode	retention time (min)	precursor ion (m/z)	product ions (m/z)[1]	fragmentor (V)	collision energy (V)	qualifier ratio
uric acid	neg.	1.3	167.0	124.0	91	14	11.1-22.3
				69.2	91	20	
allantoin	neg.	17	157.0	113.9	96	15	96.6-144.0
				96.9	96	15	

[1]The first listed product ion was used for quantification.

Supplementary Table 6 GC-MS parameters for carbohydrate quantification. Retention times are affected by matrix and can vary. First product ion listed was used for quantification.

analyte	retention time (min)	precursor ion (m/z)	product ions (m/z)
U-^{13}C-ribose	9.58	472.9	220 and 310
ribose	9.58	467.9	217 and 307
U-^{13}C-D7-glucose	10.81[1]	583.1	327 and 210
glucose	10.84	570.1	319 and 205
U-^{13}C-fructose	10.67	576.1	327 and 210
fructose	10.67	570.1	319 and 205
sucrose (U-^{13}C-fructose)	13.98	925.7	327 and 217
sucrose	13.98	919.7	319 and 217

[1] shift in retention time due to the deuterium isotope effect (Gathungu et al., 2020).

Supplementary Methods

Quantification of mNeonGreen-SKL fluorescence

Images from independent sections from two nodules expressing the mNeonGreen-SKL under the control of the pGlcT;a promoter and 5’-UTR were analyzed. Cell boundaries cannot be easily distinguished in the bright field images, but areas with infected cells appear smooth gray whereas areas with uninfected cells are characterized by the occurrence of numerous starch granules, that are known to occur only in uninfected cells (Atkins et al., 1984; Taté et al., 1994). This was used to separate infected from uninfected areas and quantify the associated fluorescence. The number of pixels representing fluorescence (green pixels) was counted using ImageJ Fiji converting the images into 16-bit and selecting all pixels with a color threshold between 10 and 65535 to exclude the background. The counted pixels were then divided by the total number of pixels present in the respective area and values expressed as percentage.

Supplementary References

1. Atkins, C. A., Shelp, B. J., Kuo, J., Poples, M. B. & Pate, J. S. Nitrogen nutrition and the development and senescence of nodules in cowpea seedlings. Planta **162**, 316-326 (1984).

2. Gathungu, R.M., Kautz, R., Kristal, B.S., Bird, S.S. & Vouros, P. The integration of LC-MS and NMR for the analysis of low molecular weight trace analytes in complex matrices. Mass Spectrom. Rev. 39, 35–54 (2020).

3. Taté, R., Patriarca, E. J., Riccio, A., Defez R. & Iaccarino, M. Development of Phaseolus vulgaris root nodules. Mol. Plant-Microbe Interact. **7**, 582-589 (1994).
