# Supplementary material for: A plastid carbohydrate carrier mediates ribose recycling from nucleotide catabolism and glucose export from starch degradation
Source: Nat Commun. 2025 Nov 5;16:9747. doi: 10.1038/s41467-025-65510-8 (PMC12589546; doi:10.1038/s41467-025-65510-8)
Supplement: Supplementary file 2 — Reporting Summary [file 41467_2025_65510_MOESM2_ESM.pdf]

Corresponding author(s): Claus-Peter Witte

Last updated by author(s): 26/09/25

Reporting Summary

Nature Portfolio wishes to improve the reproducibility of the work that we publish. This form provides structure for consistency and transparency in reporting. For further information on Nature Portfolio policies, see our Editorial Policies and the Editorial Policy Checklist.

Statistics

For all statistical analyses, confirm that the following items are present in the figure legend, table legend, main text, or Methods section.

n/a	Confirmed	
☐	☒	The exact sample size (*n*) for each experimental group/condition, given as a discrete number and unit of measurement
☐	☒	A statement on whether measurements were taken from distinct samples or whether the same sample was measured repeatedly
☐	☒	The statistical test(s) used AND whether they are one- or two-sided<br>*Only common tests should be described solely by name; describe more complex techniques in the Methods section.*
☒	☐	A description of all covariates tested
☐	☒	A description of any assumptions or corrections, such as tests of normality and adjustment for multiple comparisons
☐	☒	A full description of the statistical parameters including central tendency (e.g. means) or other basic estimates (e.g. regression coefficient) AND variation (e.g. standard deviation) or associated estimates of uncertainty (e.g. confidence intervals)
☐	☒	For null hypothesis testing, the test statistic (e.g. *F*, *t*, *r*) with confidence intervals, effect sizes, degrees of freedom and *P* value noted<br>*Give P values as exact values whenever suitable.*
☒	☐	For Bayesian analysis, information on the choice of priors and Markov chain Monte Carlo settings
☒	☐	For hierarchical and complex designs, identification of the appropriate level for tests and full reporting of outcomes
☒	☐	Estimates of effect sizes (e.g. Cohen's *d*, Pearson's *r*), indicating how they were calculated

Our web collection on statistics for biologists contains articles on many of the points above.

Software and code

Policy information about availability of computer code

Data collection	Leica Application Suite X (Ver. 3.7.2.22383), MassHunter Workstation Software LC/MS Data Acquisition for 6400 Series Triple Quadrupole (Ver. B.07.00), NIS-Elements Viewer (Ver. 4.20)
Data analysis	Excel 2016, GraphPad Prism 4, R software (Ver. 1.2.5042), MassHunter Workstation Software Quantitative Analysis (Ver. B.09.00), Affinity Designer 1.10.6.1665, Affinity Photo 1.10.1.1142, ImageJ Fiji

For manuscripts utilizing custom algorithms or software that are central to the research but not yet described in published literature, software must be made available to editors and reviewers. We strongly encourage code deposition in a community repository (e.g. GitHub). See the Nature Portfolio guidelines for submitting code & software for further information.

Data

Policy information about availability of data

All manuscripts must include a data availability statement. This statement should provide the following information, where applicable:

- Accession codes, unique identifiers, or web links for publicly available datasets
- A description of any restrictions on data availability
- For clinical datasets or third party data, please ensure that the statement adheres to our policy

The datasets generated and analyzed as part of this study are included in this article (and its supplementary information files). Mass spectrometry raw data can be supplied upon request. Source data are provided with this paper. Accession codes: Arabidopsis: pGlcT, At5g16150 [https://www.uniprot.org/uniprotkb/B9DGP1/entry]; pGlcT2, At1g05030 [https://www.uniprot.org/uniprotkb/Q0WVE9/entry]; RBSK, At1g17160 [https://www.uniprot.org/uniprotkb/A1A6H3/entry]; MEX1,

At5g17520 [https://www.uniprot.org/uniprotkb/Q9LF50/entry]. P. vulgaris (v2.1 annotation): pGlcT;a, Phvul.004G061900) [https://phytozome-next.jgi.doe.gov/report/gene/Pvulgaris_v2_1/Phvul.004G061900]; pGlcT;b, Phvul.008g007500 [https://phytozome-next.jgi.doe.gov/report/gene/Pvulgaris_v2_1/Phvul.008G007500]; RBSK, Phvul.005G070000 [https://phytozome-next.jgi.doe.gov/report/gene/Pvulgaris_v2_1/Phvul.005G070000].

Research involving human participants, their data, or biological material

Policy information about studies with human participants or human data. See also policy information about sex, gender (identity/presentation), and sexual orientation and race, ethnicity and racism.

Reporting on sex and gender	n/a
Reporting on race, ethnicity, or other socially relevant groupings	n/a
Population characteristics	n/a
Recruitment	n/a
Ethics oversight	n/a

Note that full information on the approval of the study protocol must also be provided in the manuscript.

Field-specific reporting

Please select the one below that is the best fit for your research. If you are not sure, read the appropriate sections before making your selection.

[x] Life sciences [ ] Behavioural & social sciences [ ] Ecological, evolutionary & environmental sciences

For a reference copy of the document with all sections, see nature.com/documents/nr-reporting-summary-flat.pdf

Life sciences study design

All studies must disclose on these points even when the disclosure is negative.

Sample size	For metabolite analyses, sample sizes varied from n=3 to n=7. For Arabidopsis, routinely n = 5 was chosen, which is the maximum number of biological repeats that could be handled when several genotypes and/or time points were analyzed.<br>For bean nodules, harvesting and genotyping of the possible edited nodule mutant pools was very laborious. The number of samples was highly dependent on the obtained number of identified null-mutant nodule pools.
Data exclusions	In the metabolite analysis, measurements failing the pre-established quality criteria for retention time, qualifier to quantifier ratio or with a signal to noise ratio below 10 were called “not detected”
Replication	Data shown in Fig. 1a, Fig. 1b and Fig. 2 were replicated once but there is substantial overlap in these experiments showing that core results are fully reproducible. Results from Fig. 3b was repeated two times. Uptake assays for ribose (Fig. 4) were repeated three times. The kinetics, pH-dependence of activity and inhibition assays were repeated twice. Uptake assays for glucose and fructose were repeated twice. Growth assays (Fig. 5) were repeated twice. Isolation of nodule mutants and analysis was repeated once. The study on pGlcT;a promoter activity in bean nodules was repeated twice.
Randomization	Plants of different genotypes were grown randomized and positions of individual pots frequently changed.
Blinding	After plant and nodule harvest, samples were analyzed in randomized and blind fashion.

Reporting for specific materials, systems and methods

We require information from authors about some types of materials, experimental systems and methods used in many studies. Here, indicate whether each material, system or method listed is relevant to your study. If you are not sure if a list item applies to your research, read the appropriate section before selecting a response.

Materials & experimental systems

n/a	Involved in the study
☐	☒ Antibodies
☐	☐ Eukaryotic cell lines
☐	☐ Palaeontology and archaeology
☐	☐ Animals and other organisms
☐	☐ Clinical data
☐	☐ Dual use research of concern
☐	☒ Plants

Methods

n/a	Involved in the study
☐	☐ ChIP-seq
☐	☐ Flow cytometry
☐	☐ MRI-based neuroimaging

Antibodies

Antibodies used: mouse monoclonal anti His-tag antibodies (A00186, lot No 19C001747, 1:2,500; GenScript, Netherlands) , goat polyclonal anti-mouse IgG secondary antibodies coupled to horseradish peroxidase (31430, lot No YC371618, 1:10,000; Pierce Biotechnology, USA), mouse monoclonal anti-GFP (11814460001, 1:5,000 diluted, clones 7.1 and 13.1, Roche), goat anti-mouse IgG antibody conjugated to alkaline phosphatase (A3562,1:30,000, lot No SLCP2562, Sigma)

Validation: According to the manufacturers' websites, the anti-His-antibody is suitable for ELISA; WB; IP; ICC/IF; FCM; TR-FRET; the anti-GFP antibody is suitable for WB and IP.

Eukaryotic cell lines

Policy information about cell lines and Sex and Gender in Research

Cell line source(s): n/a

Authentication: n/a

Mycoplasma contamination: n/a

Commonly misidentified lines (See ICLAC register): n/a

Palaeontology and Archaeology

Specimen provenance: n/a

Specimen deposition: n/a

Dating methods: n/a

☐ Tick this box to confirm that the raw and calibrated dates are available in the paper or in Supplementary Information.

Ethics oversight: n/a

Note that full information on the approval of the study protocol must also be provided in the manuscript.

Animals and other research organisms

Policy information about studies involving animals; ARRIVE guidelines recommended for reporting animal research, and Sex and Gender in Research

Laboratory animals: n/a

Wild animals: n/a

Reporting on sex: n/a

Field-collected samples: n/a

Ethics oversight: n/a

Note that full information on the approval of the study protocol must also be provided in the manuscript.

Clinical data

Policy information about clinical studies

All manuscripts should comply with the ICMJE guidelines for publication of clinical research and a completed CONSORT checklist must be included with all submissions.

Clinical trial registration	n/a
Study protocol	n/a
Data collection	n/a
Outcomes	n/a

Dual use research of concern

Policy information about dual use research of concern

Hazards

Could the accidental, deliberate or reckless misuse of agents or technologies generated in the work, or the application of information presented in the manuscript, pose a threat to:

No	Yes	
☒	☐	Public health
☒	☐	National security
☒	☐	Crops and/or livestock
☒	☐	Ecosystems
☒	☐	Any other significant area

Experiments of concern

Does the work involve any of these experiments of concern:

No	Yes	
☒	☐	Demonstrate how to render a vaccine ineffective
☒	☐	Confer resistance to therapeutically useful antibiotics or antiviral agents
☒	☐	Enhance the virulence of a pathogen or render a nonpathogen virulent
☒	☐	Increase transmissibility of a pathogen
☒	☐	Alter the host range of a pathogen
☒	☐	Enable evasion of diagnostic/detection modalities
☒	☐	Enable the weaponization of a biological agent or toxin
☒	☐	Any other potentially harmful combination of experiments and agents

Plants

Seed stocks	Mutant pglct-#1 (SALK051876), pglct-#2 (SALK078684), pglct2 and rbsk-1 were obtained from the Nottingham Arabidopsis Stock Centre. Bean transient CRISPR mutants in hairy roots and nodules were generated and characterized as described in the manuscript. The CRIPR mutations were not transmitted to the germline.
Novel plant genotypes	Complementation lines of pglct-2 were generated by transformation with pXCS-pGlcT-YFP (construct H391, 4 lines generated) and pXCScpmv-pGlcT (construct H392, 3 lines generated).
Authentication	The used mutants have been characterized before and the corresponding literature is cited in the manuscript. The characterization of the two transgenic lines generated in this study is shown in Supplementary Fig. S2. The used constructs are shown in the Source Data file.

ChIP-seq

Data deposition

☐ Confirm that both raw and final processed data have been deposited in a public database such as GEO.

☐ Confirm that you have deposited or provided access to graph files (e.g. BED files) for the called peaks.

Data access links<br>*May remain private before publication.*	n/a
Files in database submission	n/a
Genome browser session<br>(e.g. UCSC)	n/a

Methodology

Replicates	n/a
Sequencing depth	n/a
Antibodies	n/a
Peak calling parameters	n/a
Data quality	n/a
Software	n/a

Flow Cytometry

Plots

Confirm that:

☐ The axis labels state the marker and fluorochrome used (e.g. CD4-FITC).

☐ The axis scales are clearly visible. Include numbers along axes only for bottom left plot of group (a 'group' is an analysis of identical markers).

☐ All plots are contour plots with outliers or pseudocolor plots.

☐ A numerical value for number of cells or percentage (with statistics) is provided.

Methodology

Sample preparation	n/a
Instrument	n/a
Software	n/a
Cell population abundance	n/a
Gating strategy	n/a

☐ Tick this box to confirm that a figure exemplifying the gating strategy is provided in the Supplementary Information.

Magnetic resonance imaging

Experimental design

Design type	n/a
Design specifications	n/a
Behavioral performance measures	n/a

Acquisition

Imaging type(s) | n/a

Field strength | n/a

Sequence & imaging parameters | n/a

Area of acquisition | n/a

Diffusion MRI ☐ Used ☐ Not used

Preprocessing

Preprocessing software | n/a

Normalization | n/a

Normalization template | n/a

Noise and artifact removal | n/a

Volume censoring | n/a

Statistical modeling & inference

Model type and settings | n/a

Effect(s) tested | n/a

Specify type of analysis: ☐ Whole brain ☐ ROI-based ☐ Both

Statistic type for inference (See Eklund et al. 2016) | n/a

Correction | n/a

Models & analysis

n/a	Involved in the study
☐	☐ Functional and/or effective connectivity
☐	☐ Graph analysis
☐	☐ Multivariate modeling or predictive analysis

Functional and/or effective connectivity | n/a

Graph analysis | n/a

Multivariate modeling and predictive analysis | n/a
